# Intravitreal injection associated rhegmatogenous retinal detachment: outcomes of a European analysis

**DOI:** 10.1007/s00417-021-05261-6

**Published:** 2021-07-03

**Authors:** Efstathios Vounotrypidis, Sigrid Freissinger, Matteo Cereda, Davide Monteduro, Karsten Kortuem, Siegfried Priglinger, Benjamin Mayer, Armin Wolf

**Affiliations:** 1 Department of Ophthalmology, University of Ulm, Prittwitzstr 43, D-89075 Ulm, Germany; 2 Department of Ophthalmology, Ludwig-Maximilians-University Munich, Munich, Germany; 3 Eye Clinic, Department of Clinical and Biomedical Science Luigi Sacco, Sacco Hospital, University of Milan, Milan, Italy; 4 Department of Statistics, University of Ulm, Ulm, Germany

**Keywords:** Rhegmatogenous retinal detachment, Intravitreal injection, Proliferative vitreoretinopathy, Vitrectomy, Visual outcome

## Abstract

**Purpose:**

As the number of intravitreal injections (IVI) increases annually, this study aimed to assess the anatomical and functional outcomes following rhegmatogenous retinal detachment (RRD) surgery for IVI-associated RRD (IVARD).

**Methods:**

All non-vitrectomized eyes developing IVARD since 2007 in two European vitreoretinal centers (Department of Ophthalmology, LMU Munich, Germany, and Eye Clinic Luigi Sacco, University of Milan, Milan, Italy) were included. Main outcomes were primary and secondary retinal attachment rate after surgery, rate of proliferative vitreoretinopathy (PVR), and final functional result. Ten years of incidence rates per injection were calculated for one center.

**Results:**

Fifty-two eyes of 52 patients comprised the study. Primary anatomic success rate was 83% (n = 43) and secondary 96% (n = 50). PVR was observed in all uveitic eyes (n = 3), in eyes with postoperative cystoid macular edema (n = 2), and in 8 of 9 eyes that received the dexamethasone implant (DEX). Age, number of prior injections, duration of symptoms, or time between last IVI and RRD did not show any statistically significant differences with regard to presence of PVR or not. Mean BCVA improved in 28 cases, remained stable in 16 cases, and worsened in 8 cases. The RRD incidence rate was statistically significant higher for DEX and ocriplasmin compared with that for anti-VEGF agents.

**Conclusion:**

The anatomical result after one surgical intervention seems acceptable, but the final visual outcome remains rather poor, because of the underlying macular disease. In our population, injection with DEX is associated with higher IVARD rate, presence and development of PVR, and recurrent RRD in comparison with anti-VEGF agents. 
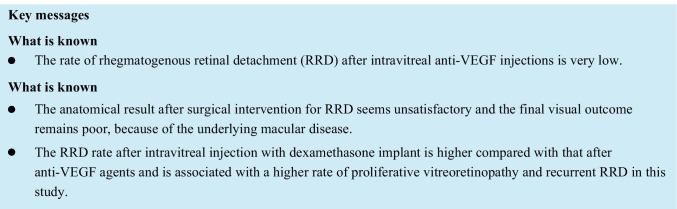

## Introduction

Intravitreal injections (IVI) of anti-vascular endothelium growth factors (anti-VEGF), steroid implants (Ozurdex® or Iluvien®), or Ocriplasmin (Jetrea®) have revolutionized the treatment of neovascular age-related degeneration (nAMD) [1–3], retinal vein occlusion (RVO) [4–6], diabetic macular edema (DME) [7–9], uveitis [10], postoperative cystoid macular edema (PCME) [11], vitreomacular traction (VMTS), or full thickness macular hole [12–15]. Whereas fewer than 2000 IVIs were administered annually in the USA in 2000, their number had soared to more than 3.0 million injections in 2016 [16]. Taking into account the increasing prevalence of AMD and diabetic retinopathy, the number of IVIs is expected to rise further globally [17, 18].

Among the various complications of IVIs [19–21], endophthalmitis and retinal detachment are the most severe and vision threatening, with rates of endophthalmitis ranging from approximately 1 in 2000–3000 injections [22, 23] and rates of rhegmatogenous retinal detachment (RRD) after IVI appearing even lower [1, 20, 24]. However, outcome and complications of RRD repair in these eyes have not yet been reported.

This retrospective explorative study has been conducted to evaluate the anatomical and visual outcome after surgical repair of IVI-associated RRD (IVARD). To our knowledge, this is the first multicenter case series including steroids and ocriplasmin that tried to figure out any effect of various factors, such as the indication for IVI, number of injections, presence of proliferative vitreoretinopathy (PVR), and duration of symptoms on the final outcome. Furthermore, RRD incidence rates over a period of ten years have been calculated for one center.

## Methods

This retrospective case series enrolled patients from two tertiary vitreoretinal centers (Department of Ophthalmology, LMU Munich, Germany and Eye Clinic, Department of Clinical and Biomedical Science Luigi Sacco, Sacco Hospital, University of Milan, Italy). The study was approved by the institutional review boards of each institution and adhered to the tenets of the Declaration of Helsinki.

A detailed search in the electronic database of each center was performed for identification of patients with a primary diagnosis of RRD that underwent IVI in the same eye in the same center. Exported data included age, gender, localization, indication for IVI, IVI agent, number of prior injections, time period between IVI and RRD, lens status, macular state, presence of proliferative vitreoretinopathy (PVR) at presentation, date and type of intervention, type of injected tamponade, and visual acuity prior to and after surgery. All patients underwent surgery for RRD repair between 15 May 2007 and 06 March 2019, and IVI was performed between 15 December 2006 and 14 October 2018. “Primary PVR” was defined as the presence of clinical signs of PVR such as starfolds before the first surgery; “secondary PVR” was considered a clinical PVR after the first surgical intervention. Overall, PVR was defined as either primary or secondary PVR during the follow-up period.

### Inclusion and exclusion criteria

Non-vitrectomized phakic or pseudophakic eyes that developed IVARD for any given diagnosis were included. No time limit was set between the time point of last IVI and RRD. A subgroup analysis of eyes having an RRD within 90 days after the last IVI was additionally performed similarly to other large studies.

### Injection

All IVIs were performed in a standard tertiary clinic setting, in a designated procedure room. Eyes were preoperatively prepared with a topical anesthetic and povidone iodine. Injection was performed with a standard 30- or 31-gauge needle in case of anti-VEGF agents (0.05 ml of Bevacizumab, Ranibizumab or Aflibercept), Ocriplasmin (0.1 ml) or recombinant tissue plasminogen activator (rt-PA; 50 µg/0.05 ml) combined with 0.5 ml of SF6 gas. The standard preloaded system was used with a 22-gauge needle for injections of the 0.7 mg slow releasing dexamethasone implant (DEX) and with a 25-gauge needle for the 0.19 mg fluocinolone acetonide implant (Ilu). Injections were performed 3.5 to 4.0 mm from the limbus. Physicians individually determined the use of subconjunctival lidocaine, the use of a bladed lid speculum, a conjunctival displacement before injection, caliper use, and the injection site.

### Surgical intervention of RRD

All eyes that developed IVARD were treated as soon as possible, based on the anatomic features of the RRD and the evaluation of a consultant ophthalmologist. The surgical approach included either scleral buckling with cryopexy or standard small-gauge pars plana vitrectomy (ppV) with laser retinopexy or a combination of both. Silicone oil or gas (C2F6, 15%) was used as primary tamponade. All cases of a recurrence of an RRD were treated with ppV.

### Outcomes

Primary outcome was the primary and secondary retinal attachment rates. Secondary outcomes included the presence and development of primary, secondary, and overall PVR and any association with indication for IVI, IVI agent, number of injections, duration of symptoms, time between IVI and RRD, and macular state. BCVA was chosen not to be the primary outcome as all the patients had functionally affected macula, and thus visual outcome after RRD repair would not be of value for the assessment of surgical results. Snellen visual acuity was converted to the logarithm of the minimum angle of resolution (logMAR) equivalent for statistical tests. Vision levels of counting fingers and hand movements were assigned visual acuity values of 2.0 and 3.0 logMAR according to previous literature [25]. Cases with pre- or postoperative BCVA of light perception were excluded from the visual outcome analysis and were reported separately.

Furthermore, the incidence rate of RRD per number of injections over a 10-year period was calculated for the Department of Ophthalmology, LMU Munich, Germany.

### Statistical analysis

SPSS statistics software package version 25 for Windows (IBM, Armonk, NY, USA) was used for statistical analysis. Collected data were tested for normal distribution by the Kolmogorov–Smirnov test. Non-parametrical tests were performed in the absence of normal distributions and t-tests in cases displaying normal distribution. Chi-square or exact Fisher’s test was applied for the evaluation of any associations between categorical variables. Analysis of variance (Kruskal–Wallis ANOVA with Dunn-Bonferroni adjustment) was performed for evaluating the effect of variables with more than three levels on the continuous variables or the final visual outcome. Overall, we followed, due to the retrospective nature of the data and the low number of PVR cases, a purely explorative analysis approach and considered a p ≤ 0.05 statistically significant.

## Results

### Demographics

A total of 52 eyes (29 right, 23 left) of 52 patients (22 females, 30 males) with a mean age of 66.2 ± 14.5 years presented with IVARD in the studied period of time. The mean duration of RRD symptoms was noted as 6.5 ± 7.8 (range: 1–30) days. At clinical presentation at the time of RRD, the majority of the patients had undergone more than three injections (mean, 4.3 ± 5.0; range, 1–26), with a mean duration between the last IVI and RRD of 11.2 ± 14.3 months (range: 0–60). Moderate to high myopia (> 6 diopters) was observed in 13 patients. The mean follow-up period was 10.7 ± 11.2 months (range: 1–35) after the surgery for IVARD. Detailed demographics are provided as online supplemental material.

The most common diagnosis for IVI was AMD (n = 13), followed by RVO (n = 12) and myopia (n = 11) with bevacizumab (n = 19), ranibizumab (n = 18), and DEX (n = 9) being the most frequently injected agents in our study population. More patients presented with macular affected RRD (n = 33) and reported symptoms over a mean of 8.7 ± 9.0 days (range: 1–30). Patients with attached macula (n = 19) presented after a shorter duration of symptoms (mean, 2.6 ± 1.5 days; range, 1–6).

### Surgery, anatomic success rates, and lens state

For primary surgery, 45 eyes underwent ppV, and 1 eye was treated with ppV combined with buckle surgery. A combined phacovitrectomy was performed in 17 patients (nine cases with attached macula), representing 74% of all phakic eyes (n = 23). Primary silicone oil fill (as an indicator for the presence of PVR before surgery) was chosen in 14 of 46 eyes undergoing vitrectomy. Buckle surgery alone was performed in 6 phakic cases, one being combined with pneumatic retinopexy. Overall, primary reattachment was achieved in 4 of 6 eyes with non-combined buckling surgery, in 25 of 32 eyes that received gas fill and in 14 of 14 eyes that received primary silicone oil fill, corresponding to a primary anatomic success rate (attachment rate) of 83% (43/52 eyes). Two eyes that have had previous buckle surgery and seven eyes with a prior vitrectomy with gas underwent a second surgery (100% ppV) within the first 3 months after the initial intervention. Silicone oil tamponade was necessary in 1 eye with prior buckling and in 5 eyes with previous vitrectomy; gas tamponade after primary failure was chosen in 1 eye with prior buckling and in 2 eyes with prior vitrectomy. The secondary attachment rate was 96% (50/52 eyes). Two eyes developed a third detachment at 4 and 9 months after the primary surgery, respectively. Both eyes had presented with RD plus macular involvement and signs of PVR at the time of first surgery.

Overall, only four patients of our cohort that underwent buckling surgery remained phakic at the last follow up examination. All other patients underwent concomitant cataract surgery during the first or second vitrectomy.

### Frequency of PVR and association with other factors

Primary PVR occurred in 10 out of 52 cases (19%), all with macular involving RRD. Secondary PVR was observed in 5 eyes at the time of re-detachment (10% of the cases); overall, PVR was present in 15 of 52 cases (29%) during clinical course of RD repair. By observing such low rates of PVR in our cohort, a multivariable analysis including several factors simultaneously was not possible.

Interestingly, primary PVR was observed in all 3 uveitic patients, whereas no myopic eyes showed any primary PVR. Overall, the indication for IVI showed a statistically significant association with presence of primary PVR (p = 0.014, Chi-square test). Secondary PVR occurred in 5 cases, in 3 eyes with retinal vein occlusion, 1 with neovascular AMD, and 1 with PCME.

**Fig. 1:**
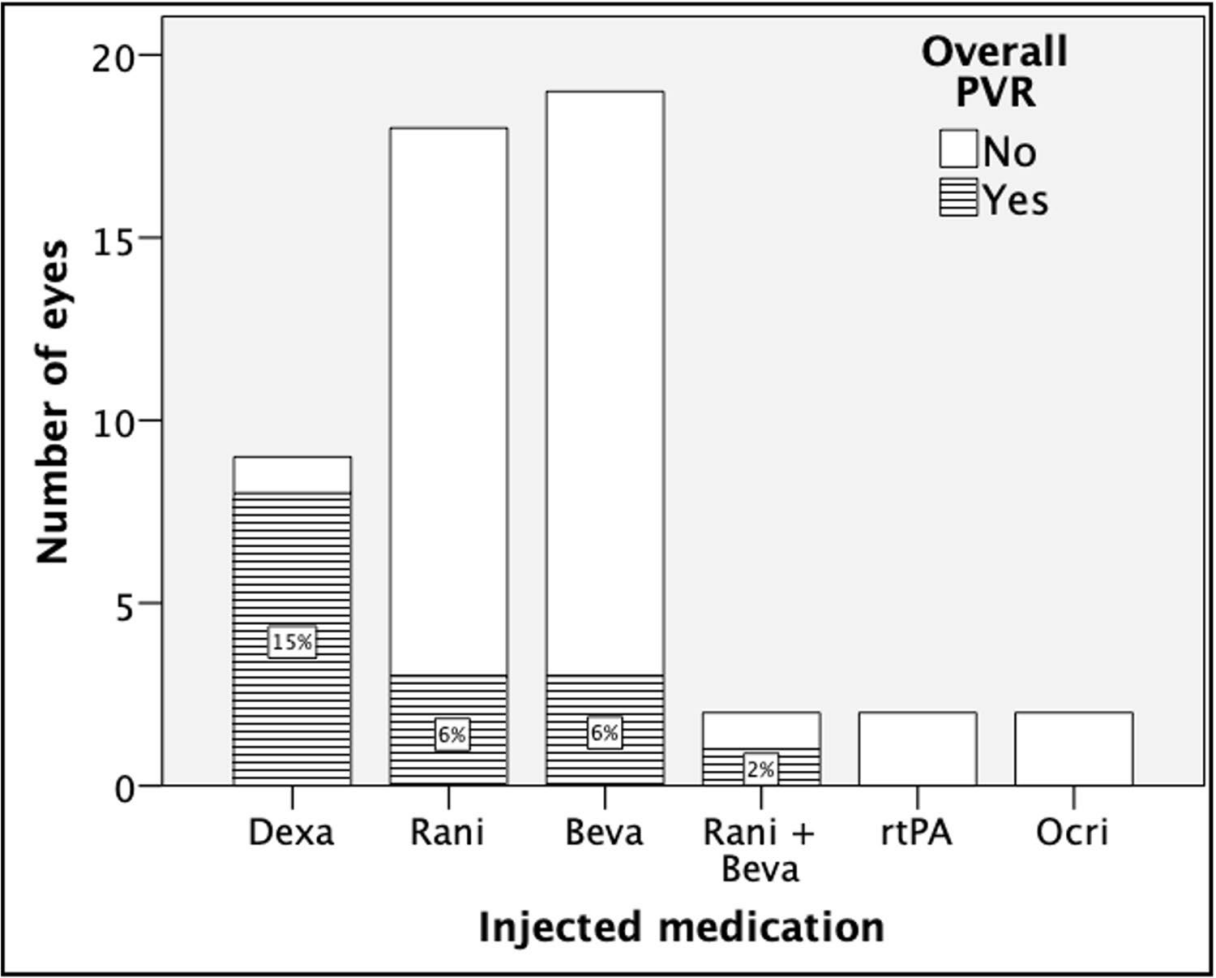
Number of eyes that developed proliferative vitreoretinopathy during the observation period with regard to the injected medication. The numbers in the bars (white boxes) correspond to their percentage. Dexa implant, Ozurdex®; Rani, Ranibizumab®; Beva, Bevacizumab®; Rani + Beva, combination of Ranibizumab® and Bevacizumab®; Rani + Afli, combination of Ranibizumab® and Aflibercept®; rtPA, recombinant tissue plasminogen activator; Ocri, Ocriplasmin®

Surprisingly, PVR, primary or secondary, was observed in eight of the nine eyes that received DEX (4 RVO, 2 Uveitis, 2 PCME), primary PVR in 5 eyes and secondary PVR in 3 eyes. Further eyes that developed primary or secondary PVR had previously received ranibizumab (3 eyes), bevacizumab (3 eyes), or aflibercept (1 eye). Frequency of overall PVR with regard to injected agent is shown in Fig. 1. Overall, all eyes that received IVI for uveitis (n = 3) or PCME (n = 2), five of 12 eyes with RVO, four of 13 with nAMD, and 1 with DME developed PVR during any time of clinical course of retinal detachment repair.

IVARD involving the macula showed a primary PVR in 10 of 23 cases, whereas no eyes without macular involvement showed any primary PVR indicating the higher association between presence of primary PVR and macular involvement (p = 0.009, Fisher’s exact test).

Age, number of prior injections, duration of symptoms and time between IVI and RRD did not differ statistically significantly between eyes that developed or did not develop PVR (p > 0.05, Mann–Whitney U in all cases). With the exception of one eye with nAMD, all other eyes with primary or secondary PVR were treated with vitrectomy and silicone oil.

### Visual acuity

Best corrected visual acuity (BCVA) was equal or better than hand movement throughout the study period in 46 of 52 eyes, whereas in six cases, a visual acuity of light perception was obtained either preoperatively or postoperatively. In particular, four cases presented with a preoperative visual acuity of light perception. In three of them, BCVA improved after surgery, and in one remained unchanged. In two other cases, BCVA worsened after the surgery to light perception. Interestingly, both eyes that developed a worse BCVA after surgery were uveitic eyes that presented with IVARD, primary PVR, and macular involvement and had previously received DEX.

**Fig. 2:**
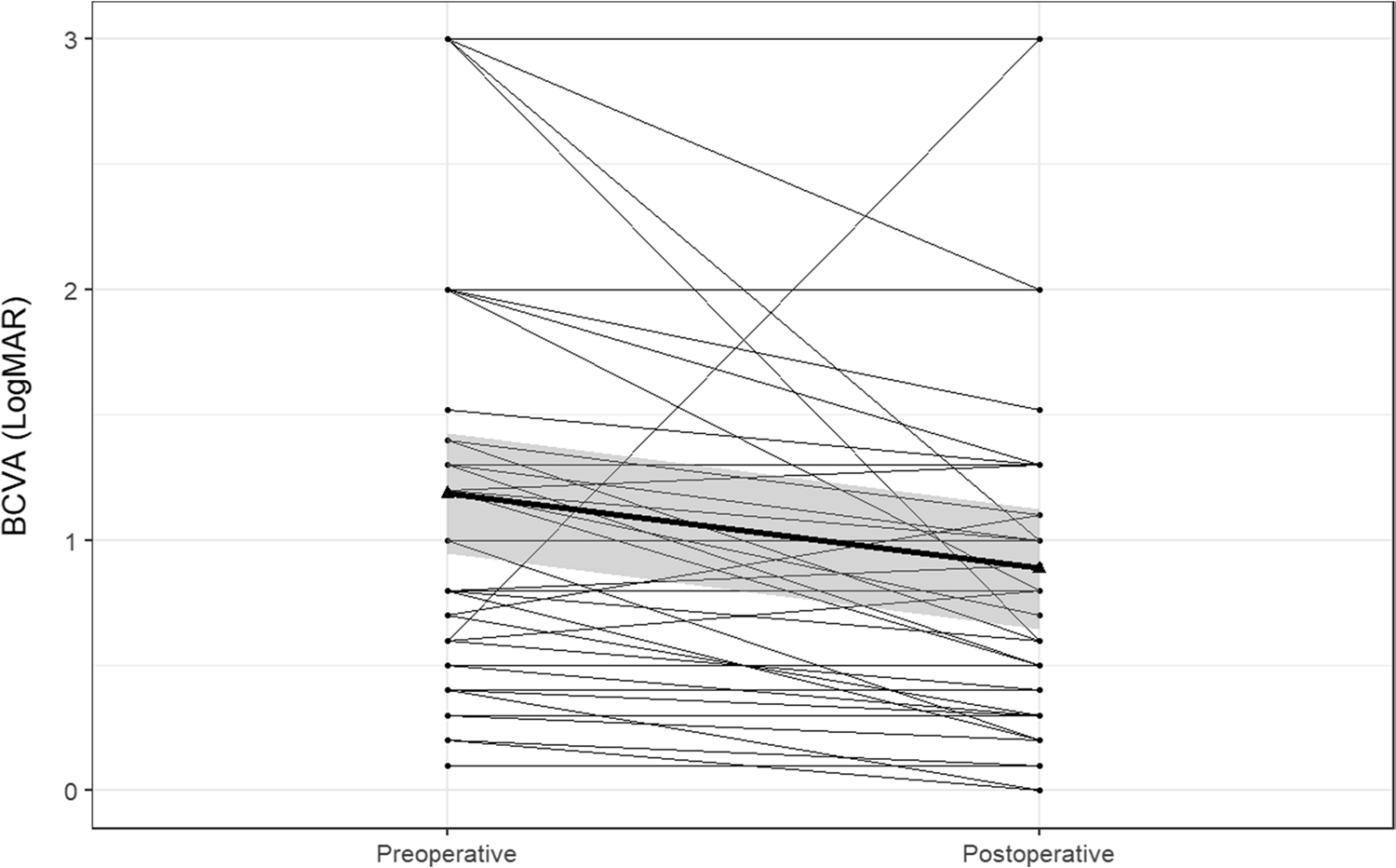
Individual trajectories of BCVA (in LogMAR) pre- and postoperatively of all patients included in the final visual outcome analysis (n = 46). The black line corresponds to the mean of all values

In the rest of the 46 eyes, mean BCVA changed from 1.19 ± 0.88 (median, 1.00; range, 0.10–3.00) to 0.89 ± 0.76 (median, 0.80; range, 0–3.00) logMAR after surgery (p < 0.0001, Wilcoxon). Four eyes that received buckling surgery only and remained phakic showed an improvement of mean BCVA from 0.43 ± 0.43 (median, 0.30; range, 0.10–1.00) to 0.23 ± 0.19 logMAR (median, 0.15; range, 0.10–0.50). In 42 eyes that underwent vitrectomy, mean BCVA improved from 1.26 ± 0.88 (median, 1.20; range, 0.20–3.00) to 0.95 ± 0.76 (median, 0.85; range, 0.00–3.00). Table 1 shows preoperative and postoperative BCVA of the 42 eyes that underwent vitrectomy with regard to preoperative macular and lens state. Figure 2 shows the individual trajectories of preoperative and postoperative BCVA of all patients that were included in the visual outcome analysis (n = 46). Overall, BCVA worsened in 8 eyes, improved in 28 eyes, and did not change more than 0.1 logMAR in 16 eyes.

**Table 1:** Mean, standard deviation (SD), median, and range of preoperative and postoperative best corrected visual acuity (in logMAR) with regard to preoperative lens and macular state of the 42 eyes that underwent pars plana vitrectomy and were included in the visual acuity outcome analysis. *BCVA* best corrected visual acuity, *On* macula preoperatively attached, *Off* macula preoperatively detached

Lens state		Phakic		Pseudophakic	
Macular state		On (n = 10)	Off (n = 9)	On (n = 6)	Off (n = 17)
BCVA preoperative (logMAR)	Mean ± SD	0.74 ± 0.85	1.63 ± 0.86	0.65 ± 0.28	1.58 ± 0.82
	Median (Range)	0.4 (0.20–3.00)	1.30 (0.70–3.00)	0.70 (0.2–1.00)	1.30 (0.30–3.00)
BCVA postoperative (logMAR)	Mean ± SD	0.32 ± 0.32	1.20 ± 0.75	0.50 ± 0.36	1.34 ± 0.76
	Median (Range)	0.30 (0.00–1.00)	1.00 (0.50–3.00)	0.45 (0.10–1.00)	1.30 (0.30–3.00)

Final postoperative BCVA did not show any significant differences with regard to the indication for IVI, injected medication, and performed surgery (p = 0.165, p = 0.105, and p = 0.133, respectively; Kruskal–Wallis). On the other hand, final postoperative BCVA was significantly lower in cases with presence of PVR or preoperatively detached macula (p = 0.011 and p = 0.0001, respectively; Mann Whitney U).

### Subgroup analysis

When we restricted the time period between IVI and RRD to 90 days, we were able to analyze 22 patients. In this group, indication for surgery included 6 RVO, 7 AMD, 2 DME, 3 myopic choroidal neovascularization (CNV), 2 VMTS, and 2 PCME cases; the applied IVIs were 5 dexamethason implants, 5 ranibizumab, 7 bevacizumab, 2 aflibercept, 1 rtPA, and 2 ocriplasmin.

In two patients, preoperative BCVA was light perception; one showed an improvement to counting finger postoperatively, and the other one showed no change after surgery. In the rest of the 20 patients, mean BCVA improved from 1.40 ± 0.97 (median, 1.3; range, 0.20–3.00) preoperatively to 1.06 ± 0.84 (median, 1.00; range, 0.00–3.00) logMAR at the last follow-up examination. Primary PVR was observed in 3 eyes (2 DEX [1 RVO, 1PCME], 1 ranibizumab [1 nAMD]) and secondary PVR in 4 other eyes (3 DEX [2 RVO, 1PCME], 1 combination of ranibizumab and bevacizumab [1 nAMD]). Interestingly, all 5 eyes that received DEX developed a primary or secondary PVR, regardless of the underlying pathology. Moreover, IVARD after ocriplasmin injection occurred within the first 2 weeks in both cases.

### Incidence rate (LMU Munich)

Assuming that all IVARDs were referred to the specific center in which the IVI had previously been performed, we calculated the incidence of RRD in the population of patients with RRD after IVI in one center only.

Following this analysis, the yearly incidence rates of RRD after IVI between 2010 and 2019 and the 10-year incidence rates with regard to the injected medication were tabulated (see Table 2). In detail, the 10-year incidence rate for ranibizumab was 0.040% (95%CI: 0.019–0.073), for bevacizumab 0.109% (95%CI: 0.054–0.195), and for the dexamethasone implant 0.214% (95%CI: 0.092 – 0.422). Aflibercept and ocriplasmin were first administered in 2013, and therefore the 7-year rates were calculated: 0.007% (95%CI: 0.000–0.039) and 1.527% (95%CI: 0.185–5.515), respectively. Ocriplasmin showed by far the highest incidence rate of RRD after IVI, and both cases occurred within the first 2 weeks after the injection.

**Table 2:** Number of RRD (in parentheses) and injections per year with regard to injected agent. The three right columns summarize the number of all injections, the number of IVARDs, and the incidence rate of IVARD per year. The bottom rows summarize the number of injections, IVARDs per injected agent between 2010 and 2019 and the incidence IVARD rate and 95%CI per medication. The last two rows demonstrate subgroup analysis data with time between last IVI and IVARD restricted to 90 days

Year	Medication							All	RRD	Yearly rate
	Rani	Beva	Afli	Ocri	Dex	Ilu	Other			
2010	642	2540	-	-	102	-		3187	0	0
2011	2272	1311	-	-	344	-		3912	0	0
2012	2709	1369	-	-	230 (2)	-		4308	2	0.0005
2013	2681 (1)	1300 (2)	444	23 (1)	235 (1)		2	4685	5	0.0011
2014	2446 (4)	955 (1)	1153	38	246 (1)	6	26	4870	6	0.0012
2015	1776 (2)	732 (1)	1807	12	291 (1)	1	61	4680	4	0.0009
2016	2258 (2)	678 (2)	2365 (1)	24	445 (2)	5	79	5854	7	0.0012
2017	2984	566 (1)	2555	19 (1)	594	13	91	6822	2	0.0003
2018	3462	350 (1)	2867	9	610 (1)	8	92	7398	2	0.0003
2019	3863 (1)	302 (2)	3266	6	639 (1)	73	130	8279	4	0.0005
Total IVIs	25,093 (10)	10,103 (11)	14,457 (1)	131 (2)	3736 (8)	106	481	50,808 (32)	32	
Incidence Rate and 95%CI (%)	0.040 (0.019–0.073)	0.109 (0.054–0.195)	0.007 (0.000–0.039)	1.527 (0.185–5.515)	0.214 (0.092–0.422)			0.063 (0.043–0.089)		
Total IVARDs (≤ 90 d)	3	6	1	2	5			17		
90 d incidence rate and 95%CI (%)	0.012 (0.003–0.035)	0.059 (0.022–0.129)	0.007 (0.000–0.039)	1.527 (0.185–5.515)	0.134 (0.044–0.312)			0.033 (0.020–0.054)		

*IVI* intravitreal injection, *RRD* rhegmatogenous retinal detachment, *IVARD* IVI-associated RRD, *d* days, *CI* confidence interval, *Rani* Ranibizumab®, *Beva* Bevacizumab®, *Afli* Aflibercept®, *Ocri* Ocriplasmin®, *Dex* Ozurdex®, *Ilu* Iluvien®

The observed incidence rates of RRD in the Department of Ophthalmology, LMU Munich, were significantly higher for ocriplasmin (risk difference: + 1.5264%; 95%CI: 0.4192–5.3955) and dexamethason implant (risk difference: + 0.2136; 95%CI: 0.1077–0.4214) in comparison with anti-VEGF agents (p < 0.0001 for ocriplasmin versus all other anti-VEGF agents and p = 0.0001 for dexamethason versus all other anti-VEGF agents; two sample z-test).

In the subgroup analysis, the incidence rates of RRD after anti-VEGF agents were 0.012% (95%CI: 0.003–0.035) for ranibizumab, 0.007% (95%CI: 0.000–0.039) for aflibercept, and 0.059% (95%CI: 0.022–0.129) for bevacizumab (ranibizumab vs bevacizumab (risk difference, -0.047%; 95%CI: -0.0844 to -0.0105); ranibizumab vs aflibercept (risk difference, + 0.005%; 95%CI: -0.016 to 0.026); aflibercept vs bevacizumab (risk difference, -0.052%; 95%CI: -0.095 to -0.010); p = 0.0118, p = 0.6314, and p = 0.0165, respectively; two sample z-test), whereas the incidence rate of RRD after DEX injection (0.134%, 95%CI: 0.044–0.312) was significantly higher than that of all other anti-VEGF agents (0.02%, 95%CI: 0.010–0.037; risk difference, + 0.114%; 95%CI: 0.058–0.169; p = 0.0001, two sample z-test).

## Discussion

IVIs are nowadays the most common ophthalmological procedure in the daily routine. While complication rates are very low, severe and vision threatening complications such as RRD or endophthalmitis can lead to significant visual deterioration [26, 27]. With regard to anti-VEGF agents, a rate around 0.013% for RRD after IVI has been reported by several large retrospective studies in various parts of the world [24, 26].

The current bi-center retrospective study was conducted to evaluate the anatomic and visual outcome after RRD repair to treat IVI-related RRD and is the first study to include other agents, such as ocriplasmin and two steroids, namely, the slow releasing 0.7 mg dexamethasone implant and the slow releasing 0.19 mg fluocinolone acetonide implant.

The primary anatomical success rate after one surgery was 83% (43/52 eyes) and is considered comparable to current attachment rates in complex RRD surgery cases [28, 29]. However, it increased to 96% (50/52) after the second surgery. The unexpectedly high rate of silicone oil fill needed in our cases (with both of the centers encouraging silicone oil fill only in complex cases) can be taken as an indicator for the more than average complexity of these cases.

Overall, mean BCVA improved after surgery in the majority of the eyes with the exception of eyes with uveitis. Myopic eyes achieved the best BCVA after surgery. This might be because of the shorter duration of symptoms in this group (mean of 2.36 days), reflecting the fact that these patients may be more prone to visit an ophthalmologist in case of RRD symptoms. Moreover, eyes with myopic CNV tend to restore a good visual function upon first anti-VEGF treatment as compared with other entities such as nAMD [30]. Additionally, IVI-related RRD after ocriplasmin treatment demonstrates a very good BCVA increase after RRD repair. We consider that these patients were extensively informed about RRD risk after IVI with ocriplasmin and showed a similar short duration of symptoms of 2.5 ± 0.7 days, as in myopic patients. Overall, a short duration of RRD is known to improve the final visual outcome [31].

Eyes undergoing silicone oil fill showed a lower increase of BCVA after successful RRD repair. This fact reflects not only the worse preoperative BCVA, but also the higher complexity of these cases, including macular involvement and the presence of PVR.

PVR was observed in all uveitic eyes and eyes with PCME, whereas no PVR was observed in any myopic eyes. A possible explanation is the preexistent inflammation in uveitic eyes and in eyes with PCME, both of which increase the risk of PVR. Interestingly, eight of the nine eyes who developed RRD after the application of DEX showed a primary or secondary PVR and received silicone oil fill. Due to the small number of cases in our population, one can make only assumptions about the possible explanation of this observation. However, two major factors differ between DEX and other injections. One is the larger sclerotomy and the larger needle (22-gauge needle) used for DEX injection, which leads to a higher possibility for vitreous leakage. The second is the different underlying disease spectrum, as more inflammatory eyes (i.e., uveitic, PCME) or eyes with disrupted blood retinal barrier (such as in RVO) are treated with DEX [6, 9, 10]. Since it remains unclear if the dexamethasone implant may impact the development of primary or secondary PVR in these individual cases or if the underlying disease is responsible for this finding, both factors require further investigation for drawing concrete conclusions regarding any association between them and development of PVR. Up to now, the possible adjunct effect of DEX in the treatment of PVR retinal detachment has not been confirmed [32], and the role of DEX in the development of PVR is still not adequately investigated [33]. In our population that had previously received DEX, the implant was removed during primary RRD surgery, and we cannot rule out that this may have resulted in a sudden decrease of steroids leading to a more intense blood retina breakdown that promoted secondary PVR in these eyes.

Overall, the observed incidence rate of RRD after DEX injection or ocriplasmin was significantly higher in comparison to the other anti-VEGF agents. Previous studies have shown varying rates of RRD after DEX [6, 10, 34]. Lowder et al. reported 2 RRD cases in 153 DEX injections in uveitic eyes after a follow-up of 26 weeks [10], Haller et al. 1 case in 2512 DEX injections after a follow-up of 52 weeks in eyes with RVO [6], and Rajesh et al. a rate of 0.03% in 6000 DEX injections with varying indications (52% DME) and a different follow-up [34]. The observed rate in this study lies between the previously reported rates. As for ocriplasmin, prior studies have already indicated RRD as a possible complication [6, 13, 15, 35], because ocriplasmin is associated with vitreous liquefaction, posterior vitreous detachment, and reduced adherence between the retina and RPE as a result of its proteolytic effect [36]. Our study results are in agreement with these findings, as both RRD cases presented within the first 2 weeks after the injection, supporting this causality. With regard to the incidence rate of RRD after anti-VEGF agents, we observed a very low rate for all three anti-VEGF agents. These data are in accordance with those in the literature and, despite some differences, reflect the published rates after each type of injection [24, 26]. The incidence rate of RRD after the 0.19 mg fluocinolone acetonide implant (Iluvien) was zero. However, this fact relies on the very small number of injections in our cohort and does not reflect a long-term incidence rate of RRD after such injections. Therefore, this finding cannot be generalized, and no comparisons with other intravitreal injected agents were performed.

The retrospective design of the study and the small sample size are the major limitations of our data. Furthermore, while we cannot be sure that all the patients that developed IVARD in each clinic were referred to the same clinic, we think that our data rather underreport the incidence rate of IVARD. Nevertheless, we have enrolled all cases over an extensive period of time in two different centers and included ocriplasmin and cortisone implants in our study. Furthermore, we have conducted a subgroup analysis by reducing the time between IVI and RRD to 90 days to make our results comparable with the largest published study in the USA and calculated the 10-year incidence rates for one large vitreoretinal center in central Europe.

In conclusion, the anatomical result after one surgical intervention in cases of IVARD is graded as acceptable for such complex cases, but the final visual outcome remains rather poor, most probably because of the underlying macular disease. Furthermore, the presence and development of PVR seem to occur unexpectedly often in these cases. In particular, eyes with IVARD in the need for silicone oil fill remain behind expectations concerning the functional development.

The RRD rate after IVI with dexamethasone implant seems to be higher compared with that after anti-VEGF agents and is associated with a higher rate of PVR presence and recurrent RRD in our population. The rate of RRD after IVI with fluocinolone implant needs to be further determined in the future in a larger population.

## Supplementary Information

Below is the link to the electronic supplementary material.

Supplementary file1 (DOCX 16.0 KB)
